# Impacts of Diabetes Self-Management Education and Support Programs in Hispanic Church Settings: A Cluster-Randomized Trial Comparing Faith-Based and Faith-Placed Approaches

**DOI:** 10.3390/nu17010069

**Published:** 2024-12-28

**Authors:** Summer R. Wilmoth, Leah L. Carrillo-McCracken, Bradley Wilhite, Meixia Pan, Deborah Parra-Medina, Erica T. Sosa, Ramon Reyes, Meizi He

**Affiliations:** 1 Department of Public Health, College for Health, Community and Policy, The University of Texas at San Antonio, San Antonio, TX 78249, USA; summer.wilmoth@utsa.edu (S.R.W.); erica.sosa@utsa.edu (E.T.S.); 2 Barshop Institute, UT Health Science Center (UT Health) San Antonio, San Antonio, TX 78229, USA; panm@uthscsa.edu; 3 Center for Health Equity, Department of Family Medicine, School of Medicine, University of Colorado Anschutz Medical Campus, Aurora, CO 80045, USA; 4 Bandera Family Health Care Research, San Antonio, TX 78249, USA; drreyes@banderafamily.com

**Keywords:** diabetes self-management education support, faith-based, church-based health, Hispanic health disparities, diabetes self-care activities

## Abstract

**Background/Objectives:** This study aimed to adapt evidence-based diabetes self-management education and support (DSMES) into a faith-based (FB) context for Hispanic communities and compare its effectiveness to a faith-placed (FP) approach using the church as a venue for DSMES delivery. **Methods:** A cluster-randomized trial was conducted among adults with type 2 diabetes from predominantly Hispanic churches. The churches were assigned to either the FB Group (nine churches, n = 146) or the FP Group (seven churches, n = 125). The FB Group, led by trained lay health leaders, received a health sermon, a six-session DSMES program, and a seven-session Healthy Bible Study. The FP Group, led by outside health professionals, received the same six-session DSMES and a seven-session partial attention control curriculum. Key outcomes, including hemoglobin A1c (HbA1c), waist circumference (WC), diabetes distress, self-care activities, and self-efficacy, were assessed at baseline, 6, 9, and 12 months. **Results:** The FB Group had lower HbA1c levels than the FP Group at 6 months (−0.3%, *p* < 0.01), with no within-group differences post-intervention. No significant between-group differences were found for other outcomes. Within-group comparisons from baseline showed that both groups reduced WC at 9 and 12 months. Both groups showed reductions in diabetes distress and increased self-efficacy at all time points post-intervention (*p* < 0.05). The FB Group increased self-care scores at all time points post-intervention, while the FP Group increased at 9 and 12 months. **Conclusions:** DSMES can be effectively delivered in church settings by trained lay leaders or health professionals in Hispanic communities. Adding a spiritual dimension to DSMES may enhance outcomes.

## 1. Introduction

Type 2 diabetes (T2D) is a major health concern in the United States (US), where 37 million adults have been diagnosed with diabetes and more than one-third of the US population with prediabetes [1]. Hispanics are 2–3 times more likely to develop T2D and related complications compared to non-Hispanic White individuals [2,3]. This increased risk is attributed to a combination of genetic, environmental, and social factors. For instance, certain genes more prevalent in Hispanic populations are linked to a higher risk of developing diabetes [4]. Higher rates of obesity and insulin resistance are more common among Hispanics [2]. Social determinants of health, including limited access to healthcare, lower socioeconomic status, and cultural factors, also contribute to the higher prevalence of T2D in this population [2].

T2D requires on-going self-management to prevent complications, and diabetes self-management education and support (DSMES) is crucial to help individuals adopt healthy behaviors to manage their conditions [5]. Despite the proven benefits [6,7], only 5% of Medicare and 6.8% of private insurance patients with diabetes receive DSMES [8]. Access to DSMES services may be particularly constrained for Hispanic patients, who may lack health insurance coverage or a regular source of primary care [9]. Financial and linguistic obstacles may further hinder Hispanic patients’ access to DSMES in primary care [9]. To address these obstacles and enhance service utilization, DSMES programs should be extended to the community setting, beyond primary care, especially for Hispanic populations [9].

Churches could serve as promising community settings for DSMES programming, given that a majority of Hispanics identify with the Catholic/Protestant faith [10]. Health promotion programming at church can take either a “faith-placed” (FP) or “faith-based” (FB) approach. The FP approach enables health professionals from outside the congregation to utilize the church as a venue for program delivery, which is a common practice for health promotion programming [11]. In contrast, the FB approach incorporates spirituality into the program and is often delivered by trained church lay leaders [12]. Research indicates that spirituality plays a significant role in positively influencing diabetes outcomes [13]. Integrating a spiritual dimension into health promotion programming fosters the adoption of healthy behaviors for a higher purpose, potentially leading to greater and more sustainable behavior changes [14]. Furthermore, church is a place where congregants serve one another by using their gifts for a meaningful purpose. Training church congregants as lay health leaders has been utilized for health program delivery in Hispanic communities [15].

The primary objective of the present study was to adapt evidence-based DSMES programming into a faith-based context to address the needs of Hispanic faith community members. We aimed to investigate whether such an FB DSMES intervention yields more sustainable intervention effects on diabetes outcomes as compared to a more traditional FP approach, which utilizes the church solely as a venue for DSMES delivery. We hypothesized that both FB and FP participants would demonstrate improvements in blood glucose control, reflected by a lower glycated hemoglobin (HbA1c) level at 6 months from baseline; however, only FB participants would maintain a lower HbA1c level at 9 and 12 months from baseline. A cluster-randomized trial with repeated measures at baseline, 6, 9, and 12 months was conducted to test the study hypothesis. This paper presents the intervention effects of both the FB and FP DSMES approach on diabetes-related outcomes.

## 2. Materials and Methods

### 2.1. Study Design

A cluster-randomized trial with repeated measures was conducted between 2017 and 2020 among church congregants with self-reported T2D in predominantly Hispanic churches affiliated with Christian denominations in San Antonio, Texas, United States. Using a two-arm design, 17 churches were randomly assigned to either the FB (***n*** = 9) or FP (***n*** = 8) intervention arm. Intervention activities were implemented over 14 consecutive weeks. The primary outcome was HbA1c level. Secondary outcomes included waist circumference (WC), body weight, diabetes distress, diabetes self-care activities, and diabetes self-efficacy. Data were collected at baseline, 6, 9, and 12 months during the study period. The study was reviewed and approved with a full review by the Institutional Review Board at the Principal Investigators’ home institution and is registered with Clinicaltrial.org. Written informed consent was obtained prior to data collection. Detailed study protocol has been published elsewhere [16].

### 2.2. Study Population

#### 2.2.1. Church and Participant Eligibility

Churches with a predominantly Hispanic (60%) congregation and at least 20 adults with self-reported type 2 diabetes (T2D) were eligible to take part in the study. Participants were adults aged 21 and older with self-reported T2D. Pregnant women were excluded from the study.

#### 2.2.2. Church and Participant Recruitment

Recruitment occurred at two levels, i.e., church and participant level. At the church level, churches in predominantly Hispanic communities were invited to take part in the study via a variety of methods (e.g., mass email, mailers, and phone calls). Churches were screened for eligibility before taking part in the study. At the participant level, congregants in the enrolled churches were recruited via weekly promotions at churches (e.g., church announcements, social media posts, and program flyers).

#### 2.2.3. Randomization and Allocation

Randomization occurred at the church level. Churches were matched by denomination (Catholic and Protestant churches) and church size (small, medium, and large). As recruitment was an on-going process, churches were randomized 1:1 by church size. Church leaders were informed of their church randomization status prior to data collection.

### 2.3. Intervention Group: Faith-Based Intervention

The FB intervention took place over the course of 14 consecutive weeks and was delivered by trained Church Lay Health Leaders from each participating church. FB participants took part in a Program Kickoff event with a Health Sermon, the 6-session Self-Management Resource Center Diabetes Self-Management Program [17], and a 7-session Healthy Bible Study. The FB intervention employed a train-the-trainer model. Each FB church identified 2–3 congregation members, with at least one person having diabetes, to be trained to become Church Lay Health Leaders. Each Church Lay Health Leader received 40 h of in-depth, hands-on skills training in Self-Management Resource Center’s Chronic Disease Self- Management Program curriculum, Diabetes Self-Management Program [18], and the Healthy Bible Study curriculum. These training sessions were offered on the weekend and were delivered by research staff who were certified Self-Management Resource Center Diabetes Self-Management Program Master Trainers [18]. Below are brief descriptions of the FB intervention components.

#### 2.3.1. Program Kickoff Event and Health Sermon

Participants first attended a brief presentation from the research team with respect to diabetes basics and FB study procedures. Additionally, participants received a Health Sermon from the church Pastor or Priest to increase their attentiveness to caring for one’s physical health as an opportunity for spiritual growth and a means to honor God.

#### 2.3.2. Self-Management Resource Center Diabetes Self-Management Program

The Self-Management Resource Center Diabetes Self-Management Program is a research-tested, self-management program for diabetes that is recognized as a Diabetes Support Program by the American Diabetes Association; and it has been approved as part of recognized Diabetes Education Programs by the American Diabetes Association and the Association of Diabetes Care and Education Specialists [18]. In the FB intervention, the six weekly Self-Management Resource Center Diabetes Self-Management Program lessons were delivered by trained Church Lay Health Leaders. During the session, participants made weekly action plans, shared experiences, and helped each other solve any problems encountered [17].

#### 2.3.3. Healthy Bible Study

The research team, in consultation with a Protestant pastor and a Catholic priest, created seven weekly Healthy Bible Study sessions. These sessions, facilitated by Church Lay Health Leaders, emphasized the link between physical and spiritual health. Each session included scripture readings, health messages, reflective questions, handouts, and take-home activities. The topics covered were Our Body, God’s Holy Temple; Strengthen God’s Holy Temple through Physical Activity; Maintain God’s Holy Temple through Eating the Right Foods; Sustain God’s Holy Temple through a Healthy Plate; Avoid Gluttony for the Glory of God; Creative Cooking for the Glory of God; and Glorify God’s Temple.

### 2.4. Comparison Group: Faith-Placed Active Control

The FP intervention also took place over 14 consecutive weeks and was delivered by outside health professionals. FP participants took part in a Program Kickoff event, the 6-session Self-Management Resource Center Diabetes Self-Management Program, and 7-session community health and safety curriculum as a partial attention control intervention.

#### 2.4.1. Program Kickoff Event

Participants attended a brief presentation from the research team with respect to diabetes basics and FP study procedures. No Health Sermon was delivered.

#### 2.4.2. Self-Management Resource Center Diabetes Self-Management Program

The same Self-Management Resource Center Diabetes Self-Management Program was also utilized to help participants manage their diabetes. However, program delivery differed from the FB arm. Two certified facilitators from the local public health department implemented the self-management curriculum for six consecutive weeks in the church setting.

#### 2.4.3. Partial Attention Control Intervention

To ensure that participants in both arms received an equal amount of intervention exposure (attention), participants received seven weekly sessions of a first aid and safety curriculum, unrelated to diabetes self-management, delivered by two research staff.

### 2.5. Study Measures and Data Collection

The primary outcome was HbA1c level [19], and the secondary outcomes included the following: waist circumference, body weight, diabetes distress [20,21], diabetes self-care activities [22,23], and diabetes self-efficacy [24] (Table 1). Outcome data were collected at baseline, 6, 9, and 12 months. Social demographics (e.g., gender and income) and acculturation [25] were also collected. Intervention implementation process data were tracked throughout the program.

Data collection occurred at each participating church. Bilingual research staff explained study purposes and procedures to potential subjects, invited voluntary participation, answered questions, and obtained informed consent. Trained research staff performed HbA1c testing following standard finger-prick protocol. Anthropometric measurements were taken in a private area to protect participants’ privacy. Other secondary outcomes were self-identified and reported by participants on a self-administered questionnaire with assistance from trained research staff. Written informed consent was obtained for all study participants prior to data collection. A detailed study protocol has been published elsewhere [16]. Participants received a USD 10 gift card at each data collection point as a token of appreciation.

### 2.6. Statistical Analysis

Data were analyzed using IBM SPSS 28 software, with the level of significance for all statistical tests set at 0.05. The study followed the intent-to-treat analysis principle. Baseline characteristics of participants were described and compared between the FB and FP Group using the Student *t*-test for continuous variables and the Pearson Chi-square test for categorical variables.

The analyses accounted for the clustering of repeated measurements (baseline, 6-, 9-, and 12-month) within participants, who were nested within churches. Linear Mixed Models were employed to determine the intervention effect on key outcomes. These models included the outcome variable as the dependent variable, with treatment assignment (FB vs. FP) and time point (baseline, 6, 9, and 12 months) as fixed factors. Random intercepts were used to account for the multiple levels of nesting. All models controlled for covariates, including gender, age, and corresponding baseline outcome variables.

Intervention effects on key outcomes were reported as between-group mean differences over time points. To observe differences between groups at multiple time points, an interaction term for treatment assignment by time point was included in the analyses. Additionally, we present within-group post-intervention mean differences (at 6, 9, and 12 months) from baseline to assess our hypothesis that both the FB and FP Group would show improvement in diabetes-related outcomes at 6 months from baseline and to determine whether only the FB Group would sustain the improvement at 9 and 12 months from baseline.

## 3. Results

### 3.1. Study Participant Enrollment, Retention, and Program Attendance

**Figure 1:**
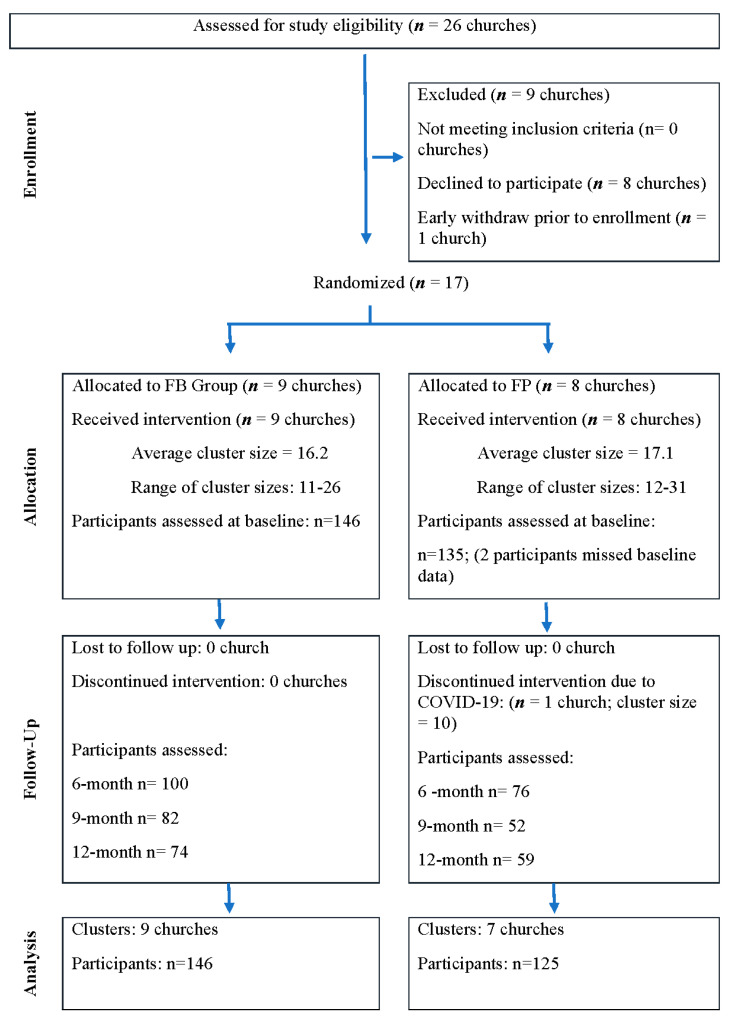
Study flow chart.

Twenty-six churches were screened for study eligibility between 2017 and 2020. Eight out of 26 churches declined participation due to concerns over intense lay leader responsibility or the inability to sign up approximately 20 individuals with diabetes for the study. Although 18 churches initially agreed to participate, one uncommitted church withdrew before formal enrollment. Consequently, a total of 17 churches, comprising 283 participants, were enrolled in the study during the recruitment period. Among these, nine churches were randomly assigned to the FB Group and eight to the FP Group. One church did not complete the assigned intervention activities due to the onset of the COVID-19 pandemic. As such, 16 churches with 271 participants were included in data analysis (Figure 1. Study flow chart). Out of 14 program sessions, average participant attendance was 9.2 sessions in the FB Group and 7.0 sessions in the FP Group (F = 20.1 *p* < 0.01). Participant attendance is an intervention implementation process measure that reflects the level of participation in the intervention activities.

### 3.2. Characteristics of Study Participants

Study participant characteristics at baseline are shown in Table 2. Study participants’ mean age was 62.2 (±12.7) years, and a majority of participants were female, of Hispanic origin, married or living with a partner, and affiliated with Catholic churches. Compared to the FP Group, the FB Group had a higher proportion of participants of Hispanic/Latino origin (91% versus 84%, Chi-square = 7.5, *p* < 0.05), a lower proportion of affiliating with Catholic churches (64% versus 88%, Chi-square = 42.5, *p* < 0.01), and a higher Problem Areas in Diabetes Score (25.6 versus 22.9, *t* = 4.3, *p* < 0.05). No statistical differences were found between groups for the rest of the baseline characteristics.

### 3.3. Intervention Effects

Table 3 illustrates the effectiveness of the FB and FP DSMES interventions on diabetes-related outcomes, including HbA1c levels, anthropometric measures, Problem Areas in Diabetes Scores, Summary of Diabetes Self-Care Activities Scale Scores, and Diabetes Self-Efficacy Scale Scores.

#### 3.3.1. HbA1c

For the primary outcome HbA1c, there is no time and group interaction (F = 1.3, *p* = 0.23). Between-group comparisons show that the FB Group had a lower HbA1c level than the FP Group at 6 months (−0.3% [95% CI, −0.5, −0.1], *p* < 0.01). No post-intervention within-group difference was observed from baseline in either the FB or the FP Group.

#### 3.3.2. Anthropometric Measures

Among overweight and obese (baseline BMI > 25) participants, there was a treatment and time interaction for waist circumference (F = 6.9, *p* < 0.01) but with no significant difference between groups at any given time point. Within-group comparisons showed reductions in waist circumference from baseline at 9 and 12 months for both groups. No between- or within-group differences were observed for bodyweight.

#### 3.3.3. Problem Areas in Diabetes Score

There is a treatment and time interaction (F = 20.1, *p* < 0.01) but with no significant difference between groups at any given time point. Within-group pairwise comparisons showed sustained reductions in Problem Areas in Diabetes Scores from baseline at 6, 9, and 12 months for both the FB Group (*p* < 0.05) and the FP Group (*p* < 0.05).

#### 3.3.4. Summary of Diabetes Self-Care Activities Scale Score

There was a treatment and time interaction (F = 8.5, *p* < 0.01) with no significant difference between groups at any given time point. Within-group pairwise comparisons showed that the FB Group significantly increased Summary of Diabetes Self-Care Activities Scale Scores at 6, 9, and 12 months from baseline, while the FP Group significantly increased their scores at 9 and 12 months.

#### 3.3.5. Diabetes Self-Efficacy Scale Score

There was a treatment and time interaction for Diabetes Self-Efficacy Scores (F = 8.6, *p* < 0.001) but with no significant difference between groups at any given time point. Within-group pairwise comparisons showed that both the FB and FP Groups had significantly increased Diabetes Self-Efficacy Scale Scores at 6, 9, and 12 months from baseline.

## 4. Discussion

The present study is the first of its kind to assess the effectiveness of an FB approach and a commonly used FP approach in DSMES delivery on diabetes-related outcomes using an RCT design. The findings revealed that the FB Group had a higher program attendance rate than the FP Group. The FB Group also had slightly lower HbA1c level compared to the FP Group at 6 months. Both the FB and FP Group showed favorable changes from to baseline, including reductions in waist circumference at 9 and 12 months, decreased diabetes distress scores, and increased self-efficacy scores at all time points post-intervention. Additionally, the FB Group showed increased self-care scores at all time points post-intervention, while the FP Group showed increases at 9 and 12 months. These results suggest that churches are indeed effective venues for delivering DSMES through either an FB or FP approach within predominantly Hispanic communities. Furthermore, integrating spirituality into secular DSMES interventions appears to offer additional benefits as evidenced by improved diabetes self-care behaviors and reduced HbA1c levels in the short term.

We initially hypothesized that both the FB and FP Groups would achieve lower HbA1c levels at 6 months from baseline, with only the FB Group maintaining this improvement. However, our study results did not support this hypothesis. Nevertheless, between-group comparisons revealed that the FB Group had lower HbA1c levels than the FP Group at 6 months, but this difference was not observed at 9 and 12 months. This may be attributed to the lack of on-going DSMES group activities after our 14-week intervention. Indeed, DSMES research conducted in church settings has yielded inconsistent results regarding HbA1c levels. For example, our own pilot study 6-weekly session DSMES using an FP approach resulted in a 0.7% HbA1c reduction at three months in Hispanic church settings [26]. An 8-month intensive DSMES intervention using an FP approach in Black churches observed a 0.4% reduction in HbA1c levels at 8 months (i.e., the end of the intervention) but not during the 12-month follow-up [27]. A 6-weekly FB DSMES intervention including prayers and delivered by lay church leaders led to a significant reduction in HbA1c levels at 3 months but not at 6 months among low-income Hispanic adults with diabetes [15]. The inconsistent results in HbA1c may be attributed to the lack of ongoing intervention at the time of the HbA1c assessment. The research showed that continued weekly group sessions was a critical strategy in sustaining HbA1c improvements at the 12- and 18-month follow-up [28]. Nevertheless, our FB approach resulted in a lower HbA1c levels at 6 months than the FP approach. Future DSMES programming in church settings should explore strategies to offer ongoing support to sustain intervention effects on HbA1c beyond 6 months.

Our study showed that the church setting is a promising venue for DSMES delivering, whether through an FB or FP approach. Churches’ social support systems make them ideal for health programing, offering essential tangible, informational, socioemotional, and spiritual support [11]. Congregants are a significant source of reciprocal support to one another through their mutually relationships. Church is a place where members may share feelings and hardships, display empathy, and express concern, love, and gratitude [29,30]. This type of supportive atmosphere is particularly important for individuals living with chronic conditions and is conducive for DSMES programing. In addition, spiritual support from church members and leaders allows for the sharing of personal religious experiences and the acquisition of spiritual insights and guidance. This spiritual support provides a sense of hope and acts as an important coping mechanism to help people manage their chronic conditions. Indeed, the integration of social support into DSMES delivered in Black churches led to health behaviors and positive health outcomes [31].

The integration of scripture into the DSMES FB intervention in the current study may have contributed to the more positive effect on diabetes outcomes compared to the FP Group. The integration of a spiritual dimension into health promotion facilitates the adoption of healthy behaviors for a higher purpose [14]. Health can be viewed as a dynamic reflection of one’s faith. Efforts to improve health can be seen as opportunities to honor God, potentially leading to more significant and sustainable behavior changes [14]. Spiritual support provided in a faith-based context extends further to deepen an individual’s faith, relationship with God, and daily faith practices [30]. Research shows that even for secular health programs delivered at church, spirituality is oftentimes naturally displayed through group dynamics of sharing and support [32]. For optimal impact, secular DSMES delivered in church settings could naturally incorporate scripture and spirituality into health programming. The integration of spirituality can take many forms, such as the delivery of health-related sermons, health-messaging enriched with scripture via Bible Study, the inclusion of prayer with health education, and physical activities involving religious music [31]. A number of efficacy studies have shown that programs integrating spiritual and physical health led to lower fasted glucose levels [33], reduced blood pressure [34], reduced weight, and improved eating behavior [35].

Our FB intervention utilizing trained health leaders from the church may have further enhanced DSMES program delivery. The roles of Community Health Workers in DSMES have been long recognized and proved effective in primary care and community settings for people of color, including Hispanic individuals [28,36,37]. Similarly, trained lay health leaders from faith communities can be effective intervention agents because they are viewed as trustworthy and are more likely to be sensitive to community needs. These leaders possess the rapport, skills, and cultural competence to deliver health promotion programs at church while providing support through the existing social network [38,39]. Our use of trained lay health leaders from the church for DSMES program delivery might have increased participant buy-in and sustained their engagement throughout the program, as reflected by the current study’s higher program attendance for the FB Group compared to the FP Group. DSMES programs delivered in church settings by trained church members offer a low-cost, accessible alternative to traditional DSMES delivered by health professionals in clinical settings. While the train-the-trainer model can be an effective strategy, program planners should be aware of the intensive training and time commitment required to certify lay health leaders for DSMES program delivery. This approach may not be practical in settings other than church. In the faith context, congregants regularly volunteer their time to serve others as part of their spiritual mission, which makes the train-the-trainer model feasible in church settings where mission, purpose, and commitment intersect.

There are a number of strengths associated with the current study. First, our translational study integrated spirituality with an evidence-based DSMES program for delivery through faith communities to reach underserved Hispanics withT2D and resulted in positive diabetes self-management outcomes. Second, the current study used a cluster-randomized trial design to determine the similarities and differences of the FB versus the FP DSMES programming on diabetes outcomes. Such a study design may minimize bias and provide the basis for statistical analyses for rigorous assessment. Third, we employed a partial attention control strategy for participants in the FP Group to balance attention, treatment contact, and social support that may have a potential “placebo” effect associated with social–behavioral interventions. Fourth, the utilization of objective measures (i.e., HbA1c, waist circumference, and Body Mass Index) ensures a valid assessment of intervention outcomes.

Conducting a randomized trial in the church setting was not short of challenges. First, translational research conducted in real-world settings often struggles to establish a true control group with minimal or no intervention. The current study consisted of no “true control group” but an “active control group” utilizing an FP approach. The FP Group, i.e., the active control, demonstrated significant improvements across all diabetes-related parameters, with the exception of HbA1c compared to its baseline. Since both groups have a significant improvement from baseline, analyses utilizing Linear Mixed Models for RCT revealed no discernible difference between the FB and FP Groups, except for HbA1c. Second, the current study recruited individuals with self-reported diabetes, some of whom had a normal baseline HbA1c leaving little to no room for HbA1c reduction over the course of the study. Third, it was challenging to secure high participant turnout for follow-up measures at 6, 9, and 12 months after the completion of the 14-week program, despite our ongoing outreach efforts through the church and the provision of USD 10 gift card incentives at each follow-up time point. Further research should consider offering better incentives, providing home or remote visit options, or arranging transportation assistance to collect follow-up data. Fourth, the COVID-19 pandemic caused one church to discontinue intervention implementation, resulting in an uneven number of churches in the FB and FP intervention arms.

This study has its limitations. For instance, several secondary outcomes were measured using self-report instruments. Although these instruments were previously validated, not all were validated specifically in the Hispanic population. Furthermore, low turnout rates at various follow-up data collection time points may compromise the study’s validity. Although Linear Mixed Models can handle missing data under the missing-at-random (MAR) assumption, verifying this assumption is challenging. If the data do not meet the MAR assumption, it may lead to biased parameter estimates. Last, participants in this study were predominately Mexican-Americans; the results may not be generalizable to the broader Hispanic population.

## 5. Conclusions

The current study suggests that DSMES can be effectively delivered in a church setting by trained church lay leaders or outside health professionals to serve the hard-to-reach Hispanic communities disproportionately affected by T2D. This study adds to the existing literature by demonstrating that integrating scripture into secular DSMES programs may enhance program outcomes, providing a spiritual–physical-health-integrated approach that resonates with faith-based participants. Additionally, our findings underscore the importance of fostering ongoing group support after program completion to sustain improvements in diabetes outcomes. These insights contribute valuable knowledge to the field in designing and implementing effective DSMES programs in Hispanic church settings.

## Figures and Tables

**Table 1:** Study measures and instruments.

Factor and Measures	Description
HbA1c	PTS Diagnostics A1cNow^TM^ (Bayer Health Care), a rapid test with acceptable reliability and validity in the community setting via a finger-prick blood sample [19]
Waist Circumference	Waist circumference was measured at the midway between the iliac crests and the lower ribs
Body weight and Body Mass Index	Measured weight (kg)/height (m)^2^
Diabetes Distress Scores	The 20-items Problem Areas in Diabetes Scale measuring negative emotions related to diabetes (e.g., fear, anger, and frustration) [20,21]
Diabetes Self-Care Activities	15 items from the Summary of Diabetes Self-Care Activities scale measuring the frequency of performing diabetes self-care tasks [22,23], along with additional 6 items from the Perceived Dietary Adherence Questionnaire to assess participants’ adherence to dietary recommendations/diet plan over the preceding 7 days [22,23]
Diabetes Self-Efficacy	The 8-item Diabetes Self-Efficacy Scale assessing diabetes self-management efficacy [24]
Demographic profile	Items include sex, age, duration of diabetes, education level, and family income
Degree of Acculturation	The 4-item Acculturation Scale for Hispanic individuals measuring self-reported language use [25]

**Table 2:** Baseline characteristics of study participants [*n* (%)] *.

Participant Characteristics		FB (n = 146)	FP (n = 125)	Total (n = 271)	*p* Value
Age (years, mean ± SD)		60.9 ± 11.8	63.6 ± 13.6	62.2 ± 12.7	>0.05
Sex	Female	111 (76.6)	101 (78.9)	212 (77.3)	>0.05
	Male	35 (23.4)	24 (21.1)	59 (22.7)	
Hispanic, Latino, or Spanish origin	Yes	131 (91.1)	107 (83.6)	238 (88.5)	<0.05
	No	15 (8.5)	18 (16.4)	33 (11.5)	
Education	Below high school	10 (7.1)	12 (9.4)	22 (8.2)	>0.05
	High school	40 (28.4)	41 (32.0)	81 (30.1)	
	Some college/college degree	75 (53.2)	57 (44.5)	132 (49.1)	
	Graduate/Professional degree	16 (11.3)	18 (14.1)	34 (12.6)	
Employment Status	Full-time	43 (30.5)	21 (16.5)	64 (23.9)	>0.05
	Part-time	12 (8.5)	13 (10.2)	25 (9.3)	
	Unemployed	20 (14.2)	20 (15.7)	40 (14.9)	
	Retired	66 (46.8)	73 (57.5)	139 (51.9)	
Marital Status	Single/divorced/widowed	52 (36.6)	59 (46.1)	111 (41.1)	>0.05
	Married/living with partners	90 (58.9)	69 (53.9)	159 (58.9)	
Annual Family Income	Under USD 20,000	41 (30.8)	37 (32.4)	78 (31.7)	>0.05
	USD 20,000–29,999	17 (12.8)	20 (17.7)	37 (15.0)	
	USD 30,000–49,999	35 (26.3)	30 (26.5)	65 (26.4)	
	USD 50,000–99,999	36 (27.1)	22 (19.5)	48 (19.5)	
	Over USD 100,0000	4 (3.0)	4 (3.5)	8 (3.2)	
Religion Affiliation	Catholic	94 (63.5)	112 (87.5)	206 (75.2)	<0.01
	Protestant	52 (35.1)	16 (12.5)	68 (24.8)	
Acculturation Score (0–16; mean ± SD)		9.9 ± 5.4	10.3 ± 5.1	10.1 ± 5.3	>0.05
HbA1c (%; mean ± SD)		6.6 ± 1.7	6.5 ± 1.2	6.5 ± 1.5	>0.05
HbA1c (mmol/mol; mean ± SD)		49.0 ± 18.3	48.0 ± 13.1	48.5 ± 16.0	>0.05
Body Mass Index (kg/m^2^; mean ± SD)		33.5 ± 7.0	33.6 ± 8.3	33.6 ± 7.6	>0.05
Waist Circumference (cm; mean ± SD)		109.6 ± 14.6	110.7 ± 16.7	110.1 ± 15.6	>0.05
Problem Areas in Diabetes (0–100; mean ± SD)		25.6 ± 21.8	22.9 ± 20.0	24.3 ± 21.0	<0.05
**Summary of Diabetes Self-Care Activities Score** (0–147; mean ± SD)		66.1 ± 21.2	71.5 ± 22.1	68.8 ± 21.7	>0.05
Diabetes Self-Efficacy Score (0–100; mean ± SD)		64.6 ± 22.3	64.3 ± 22.8	64.5 ± 22.3	>0.05

* Case number may vary due to missing values.

**Table 3:** Diabetes outcomes over time points by groups *.

Outcomes	Adjusted Mean (s.e.)		Group Differences (95% CI)	*p* Value
	FB Group	FP Group		
**HbA1c** (%) (n = 271)				
Baseline	6.5 (0.1)	6.5 (0.1)	0 (−0.2, –0.2)	0.8
6 months	6.4 (0.1)	6.7 (0.1)	−0.3 (−0.5, −0.1)	<0.01
9 months	6.5 (0.1)	6.5 (0.1)	0 (−0.2, 0.3)	0.8
12 months	6.5 (0.1)	6.6 (0.1)	−0.1 (−0.4, 0.1)	0.3
**HbA1c** (mmol/mol) (n = 271)				
Baseline	48.0 (0.7)	47.8 (0.7)	0.2 (0.9)	0.8
6 months	46.4 (0.8)	49.6 (0.9)	−3.2 (1.2)	<0.01
9 months	47.4 (0.9)	47.0 (1.0)	0.3 (1.4)	0.8
12 months	47.3 (1.0)	48.8 (1.0)	−1.5(1.4)	0.3
**Waist Circumference** (cm) (n = 248)				
Baseline	112.0 (0.8)	111.7 (0.8)	0.3 (−1.8, 2.4)	0.77
6 months	111.3 (0.9)	110.5 (1.0)	0.8 (−1.8, 3.4)	0.5
9 months	108.8 (1.0) ^†^	107.7 (1.1) ^†^	1.1 (−1.8, 4.0)	0.4
12 months	106.5 (1.0) ^†^	109.1 (1.1) ^†^	−2.6 (−5.4, 0.3)	0.1
**Body Weight** (kg) (n = 248)				
Baseline	87.8 (0.56)	87.2 (0.59)	0.6 (−1.0, 2.2)	0.4
6 months	87.1 (0.63)	86.6 (0.67)	0.50 (−2.3, 1.3)	0.6
9 months	86.6 (0.69)	87.0 (0.77)	−0.4 (−2.4, 1.6)	0.7
12 months	86.5 (0.74)	86.1 (0.80)	0.4 (−1.7, 2.6)	0.7
**Problem Areas in Diabetes** (0–100) (n = 271)				
Baseline	24.7 (1.0)	24.1 (1.0)	0.6 (−2.1, 3.4)	0.6
6 months	16.0 (1.1) ^†^	15.9 (1.3) ^†^	0.1 (−3.3, 3.5)	1
9 months	13.9 (1.2) ^†^	16.4 (1.6) ^†^	−2.5 (−6.6, 1.1)	0.2
12 months	13.1 (1.3) ^†^	15.4 (1.5) ^†^	−2.3 (−6.3, 1.5)	0.2
**Summary of Diabetes Self-Care Activities Score** (0–147) (n = 271)				
Baseline	68.5 (1.2)	71.1 (1.3)	−2.6 (−6.1, 1.1)	0.2
6 months	76.0 (1.5) ^†^	73.6 (1.7)	2.4 (−6.5, 2.1)	0.3
9 months	77.3 (1.6) ^†^	76.5 (2.0) ^†^	0.8 (−2.1, 6.5)	0.3
12 months	78.7 (1.7) ^†^	78.4 (1.9) ^†^	0.3 (−5.0, 4.9)	1.0
**Diabetes Self-Efficacy Score** (0–100) (n = 271)				
Baseline	64.1 (1.4)	64.4 (1.4)	−0.3 (−4.1, 3.6)	0.9
6 months	72.6 (1.6) ^†^	70.0 (1.8) ^†^	2.5 (−7.6, 1.9)	0.2
9 months	73.5 (1.8) ^†^	69.9 (2.2) ^†^	3.6 (−2.5, 8.6)	0.3
12 months	76.7 (1.9) ^†^	75.2 (2.1) ^†^	1.7 (−4.0, 7.1)	0.6

* Mixed models included treatment X time point as a fixed factor, within-subject repeat measure and church intercepts as random factors, adjusted for sex, age and baseline corresponding outcome measure; ^†^ within-group comparisons with adjusted means significantly different from baseline (*p* < 0.05) by the least significant difference (LSD) pairwise comparison.

## Data Availability

The current study was registered with Clinicaltrial.org (NCT03934593) under the title “Building a Healthy Temple: A Diabetes Self-Management Support Program in Hispanic Faith Community Settings.” The data that support the findings of this study are available from the corresponding author, Dr. Meizi He, on reasonable request.
